# Human papillomaviruses 16 and 58 are distributed widely among women living in Shanghai, China, with high-grade, squamous intraepithelial lesions

**DOI:** 10.1017/S0950268818003011

**Published:** 2018-11-13

**Authors:** J. Xu, Z. Xia, L. Wang, B. Yang, Y. Zhu, X. Zhu, L. Xu

**Affiliations:** Department of Obstetrics and Gynecology, Minhang Hospital, Fudan University, 170 Xinsong Road, Minhang District, Shanghai 201199, P.R. China

**Keywords:** Epidemiology, papillomavirus

## Abstract

The distribution of human papillomaviruses (HPVs) must be understood for the control and prevention of cervical cancer. Community-based Papanicolaou and HPV DNA tests were performed on 41 578 women. The prevalences of HPV genotypes 16, 18, 31, 33, 35, 39, 45, 51, 52, 56, 58, 59, 66 and 68 were assessed. In total, 10% women were infected/co-infected by these HPVs. The infection rate increased from 7.1% in women aged ⩽30 years to 10.4% in those aged 50–60 years, and then decreased slightly to 9.9% in those aged >60 years. The HPV 16 and 58 positivity rates were significantly higher among women with high-grade squamous intraepithelial lesions (HSILs) than among those with cervicitis/negativity for intraepithelial lesion or malignancy (NILM) or low-grade SILs (LSILs). The HPV 18, 52 and 68 infection rates were significantly lower in women with HSILs than in those with NILM or LSILs. The proportion of women infected by multiple HPV strains was higher among those with HSILs. The proportions of the five most common genotypes, HPV 16, 18, 33, 52 and 58, increased with the number of co-infecting strains. HPV 16 and 58 were the high-risk HPVs in the Shanghai community and should be the focus in HPV screening and vaccination.

## Introduction

The International Agency for Research on Cancer has classified 12 human papillomavirus (HPV) genotypes (16, 18, 31, 33, 35, 39, 45, 51, 52, 56, 58 and 59) as group 1 carcinogens because of their association with cervical cancer [1]. These genotypes are thus termed high-risk HPVs (hrHPVs), and they are distributed worldwide [2]. A recent meta-analysis of data from more than 1 million women (194 studies) showed that the global prevalence of infection by the HPVs listed above was 11–12%, with considerable regional variation; the five most common HPV genotypes worldwide in women with normal cervical cytology were 16 (3.2%), 18 (1.4%), 52 (0.9%), 31 (0.8%) and 58 (0.7%) [3, 4]. Another meta-analysis of data extracted from 423 studies showed that hrHPV positivity increased from 12% in females with normal cytology to 85% in those with high-grade squamous intraepithelial lesions (HSILs); the most commonly detected hrHPVs in females with invasive cancer were HPV 16, 18 and 45 [5]. Prospective studies have shown a causal relationship between persistent hrHPV infection and the development of cervical cancer [6, 7]. This pathological linkage led to the development of HPV tests for cervical cancer screening and triage [8].

Currently, many prophylactic vaccines are now available worldwide [9]. These vaccines could be categorised into bivalent (includes HPV genotypes 16 and 18 virus-like particles (VLPs)), quadrivalent (includes HPV genotypes 6, 11, 16 and 18 VLPs) and nonavalent vaccines (includes HPV genotypes 6, 11, 16, 18, 31, 33, 45, 52 and 58 VLPs) according to the components of antigens [9–11]. Although bivalent and quadrivalent HPV vaccines have been used for about 10 years and studies confirm the substantial protective effects on the high-grade cervical abnormalities [9–11], increasing studies showed that a nonavalent vaccine could further improve the prevention of cervical HSIL in up to 90–100% of cases in Italian and French women [10, 11]. The above studies suggest a more complex epidemic trend of HPV and the distribution of HPV genotypes must be understood for more effective prevention of cervical cancer.

The most prevalent HPV genotypes, HPV infection rates, and the demographic and sociological characteristics of HPV infection vary widely by region [4, 12]. China is a developing country with a population of 1.4 billion. Definition of the most prevalent hrHPVs and their characteristics is essential for the planning of cervical cancer screening and vaccination. Unfortunately, few data are available. Here, we report on a large-scale community-based screening study performed in Shanghai. Our study revealed the characteristics of the hrHPVs distribution in the Chinese women and will hence improve vaccination strategy in local area.

## Methods

### Study design and participants

To ensure early detection and management of breast and cervical cancer, and to standardise screening and referral, the Shanghai Minhang District Health and Family Planning Commission has implemented community-based screening in Minhang District, which, based on the latest census data, has a population of 2.43 million in nine towns. Commencing in 2014, screening was implemented in four towns; more than 10 000 women are screened annually via initial Papanicolaou (Pap) and hrHPV co-testing. The follow-up for those with abnormal test results is that recommended by the American College of Obstetricians and Gynecologists 2012 Updated Consensus Guidelines for the Management of Abnormal Cervical Cancer Screening Tests and Cancer Precursors [13]. Women with abnormal Pap test results and/or who are hrHPV-positive undergo colposcopy in the central hospitals of the district. Our hospital is one such hospital; we evaluated about half of all women with abnormal Pap test results.

The study was conducted in accordance with all relevant tenets of the World Medical Association's Declaration of Helsinki. The Review Board of the Ethics Committee of Medical Research at Minhang Hospital, Fudan University (Shanghai, China) approved the study protocol (Approval Number: 2013SHMH004). Written informed consent was obtained from all participants according to the guidelines of the Chinese National Ethics Regulation Committee. All participants were informed of their right to withdraw consent personally or via relatives, caregivers or guardians. The authors assert that all procedures complied with the ethical standards of the relevant national and institutional committees on human experimentation and with the Helsinki Declaration of 1975, as revised in 2008.

### Cytological and histopathological examinations

For Pap test, a speculum is gently inserted to expose the cervix; then, a sterile cytobrush was inserted into the cervical os and gently rotated in the cervical canal and the portion of the cervix extending into the vagina to collect squamous and glandular cells. The cells were then evaluated as recommended by the manufacturer of the BD SurePath liquid-based Pap test (Becton, Dickinson and Company, Franklin Lakes, NJ, USA), and the results were reported using the Bethesda System for Reporting Cervical Cytology; Definitions, Criteria, and Explanatory Notes; Third Edition [14]. The results were classified as: cervicitis/no intraepithelial lesion or malignancy (NILM); atypical squamous cells of undetermined significance (ASC-US); low-grade squamous intraepithelial lesion (LSIL); atypical cells, cannot exclude high-grade intraepithelial lesion; HSIL; and squamous-cell carcinoma (SCC). Those with ASC-US and higher cytological grades underwent colposcopy and biopsy; four-quadrant lesion-directed biopsy samples were obtained from distinct epithelial regions in the cervical transformation zone, which turned white upon application of 5% (v/v) acetic acid using a sterile, small, Tischler biopsy forceps. For subjects with fewer than four lesions, some biopsies targeted normal-appearing, cervical transformation zone epithelium or an endocervical scraping smear was performed. All biopsies were ranked in order of lesional severity at the time of colposcopy. All specimens were processed using standard cytological and histopathological methods and were evaluated by at least two certified pathologists blinded to clinical data. The histological findings were classified as no lesion found; cervical intraepithelial neoplasia (CIN) grades 1, 2 and 3; adenocarcinoma *in situ*; SCC; and adenocarcinoma [15, 16].

### HPV genotyping

At the time of Pap testing, cervical specimens for HPV testing were collected using a sterile cytobrush simultaneously as described above, and viral DNA was extracted using QIAamp DNA Mini Kits (QIAGEN, Shanghai, China). HPV DNA evaluation and genotyping were performed using a kit provided by Huada Biotech Co. Ltd (Wuhan, China). The kit detects 12 hrHPVs (16, 18, 31, 33, 35, 39, 45, 51, 52, 56, 58 and 59), two possibly carcinogenic genotypes (66 and 68) and two low-risk strains (6 and 11). The kit employs polymerase chain reaction followed by HPV genotype-specific DNA microarray analysis. It has been approved by the Chinese Food and Drug Administration. All procedures followed the manufacturer's protocols.

### Statistical analysis

Continuous variables are presented as means ± standard deviations and categorical data are presented as frequencies (percentages). Differences between groups were evaluated using the *t* test, *χ*^2^ test or Fisher's exact probability test, as appropriate. All statistical analyses were performed with the aid of SPSS software (ver. 13.0; SPSS Inc., Chicago, IL, USA), and the significance level was set to *α* = 0.05.

## Results

### HPV prevalence by year and age

**Fig. 1.**
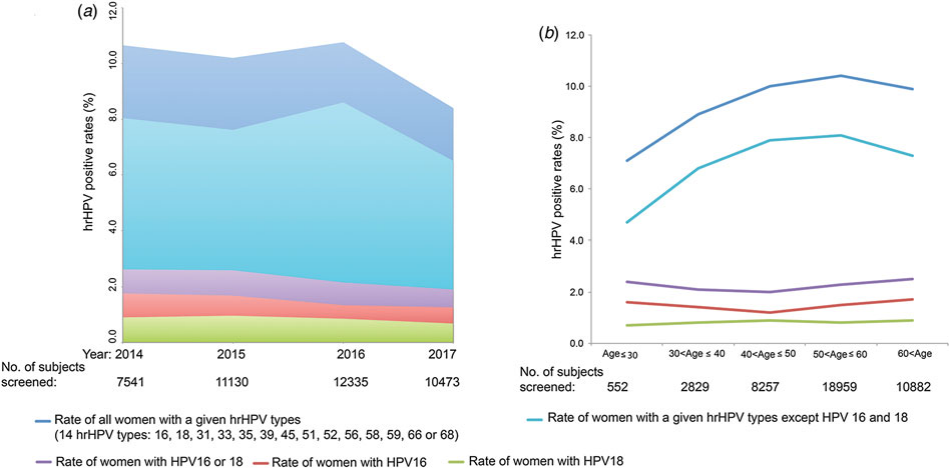
The prevalences of HPV genotypes 16, 18, 31, 33, 35, 39, 45, 51, 52, 56, 58, 59, 66 and 68 in women. (a) Overall prevalences by year. (b) Overall prevalences by age. *The English in this document has been checked by at least two professional editors, both native speakers of English. For a certificate, please see:* http://www.textcheck.com/certificate/6naFdb.

Between 2014 and 2017, a total of 41 578 women were screened using the Pap and HPV DNA tests. Of these, 4156 (10.0%) women were infected or co-infected by HPV genotypes 16, 18, 31, 33, 35, 39, 45, 51, 52, 56, 58, 59, 66 and/or 68. In 2014, 2015, 2016 and 2017, 7514, 11 130, 12 335 and 10 473 women, respectively, were co-tested. The HPV 16 and 18 positivity rates ranged from 1.3% to 1.8% and from 0.7% to 1.0%, respectively, over the 4 years. The positivity rates for serotypes 16, 18, 31, 33, 35, 39, 45, 51, 52, 56, 58, 59, 66 and 68 ranged from 6.5% to 8.0% over the 4 years (Fig. 1a). When the 41 479 subjects were pooled and stratified by age, the positivity rates for serotypes 16, 18, 31, 33, 35, 39, 45, 51, 52, 56, 58, 59, 66 and 68 were found to increase from 7.1% in women aged ⩽30 years to 10.4% in those aged 50–60 years, and then to decrease slightly to 9.9% in those aged >60 years (Fig. 1b). The rates of infection with HPV 16 (1.2–1.7%) and 18 (0.7–0.9%) were relatively low at all ages (Fig. 1b).

### Data on subjects who underwent further examination

Of all 4156 HPV-positive females, 2000 underwent colposcopy in our hospital; complete cytological and HPV data were available for 1481 of these women. Their average age was 56.3 ± 8.8 years; the numbers of women aged 21–29, 30–65 and >65 years were 11 (0.7%), 1276 (86.1%) and 194 (13.2%), respectively (Table 1). The Pap test showed that 1234 (83.3%), 58 (3.9%), 112 (7.6%), 75 (5.1%) and two (0.1%) women were of cervicitis/NILM, ASC-US, LSIL, HSIL and SCC status, respectively (Table 1). In addition, 57 (3.8%) and eight (0.5%) women, respectively, had cervical condylomas and cervical polyps, alone or in combination with the abnormal cytological changes listed above. A total of 1144 (77.2%), 278 (18.8%) and 60 (4.0%) women were infected with single, two and three or more strains, respectively (Table 1).

**Table 1.** Summary characteristics of subjects who underwent hrHPV and Pap co-testing

Age (years)	56.3 ± 8.8
Aged 21–29 years	11 (0.7)
Aged 30–65 years	1276 (86.1)
Aged >65 years	194 (13.2)
Cervicitis/NILM	1234 (83.3)
ASC-US	58 (3.9)
LSIL	112 (7.6)
HSIL	75 (5.1)
SCC	2 (0.1)
Cervical condyloma	57 (3.8)
Cervical polyp	8 (0.5)
Single hrHPV infection	1144 (77.2)
Dual hrHPV infection	278 (18.8)
Infection with three or more strains	60 (4.0)

NILM, negative for intraepithelial lesion or malignancy; ASC-US, atypical squamous cells of undetermined significance; LSIL, low-grade squamous intraepithelial lesion; HSIL, high-grade squamous intraepithelial lesion; SCC, squamous-cell carcinoma.

Age is shown as the mean ± standard deviation; the remaining data are frequencies (percentages).

### Prevalences of hrHPVs by SIL status

All HPV genotypes were common in women with cervicitis/NILM; the lowest infection rate (2.4%) was that of HPV 59 and the highest (23.9%) was that of HPV 52 (Table 2). HPV 16, 18, 52 and 58 were the most common genotypes, with infection rates >10%. The rate of infection with HPV 16 increased from 18.0% in women with cervicitis/NILM to 24.1% in those with LSILs and 54.7% in those with HSILs. The HPV 16 infection rate was significantly higher in the HSIL group than in the cervicitis/NILM, ASC-US and LSIL groups. The HPV 58 infection rate was also significantly higher in the HSIL group than in the cervicitis/NILM and LSIL groups. Although the HPV 18 infection rate was higher in women with cervicitis/NILM, ASC-US and LSILs, it was significantly lower in the HSIL group than in the other two groups. In addition, the rates of infection with HPV 58 and 52 were significantly lower in the HSIL group than in the cervicitis/NILM group.

**Table 2.** Prevalences of hrHPVs, grouped by SIL status

Genotype	Cervicitis/NILM	ASC-US	LSIL	HSIL
	(*N* = 1234)	(*N* = 58)	(*N* = 112)	(*N* = 75)
16	**222** (**18.0)**	**10** (**17.2)**	**27** (**24.1)**	**41** (54.7)***
18	**153 (12.4)**	**7 (12.1)**	**16 (14.3)**	4 (5.3)**
31	101 (8.2)	4 (6.9)	9 (8.0)	5 (6.7)
33	79 (6.4)	**7 (12.1)**	**12 (10.7)**	**8 (10.7)**
35	58 (4.7)	2 (3.4)	4 (3.6)	1 (1.3)
39	73 (5.9)	0 (0.0)	4 (3.6)	0 (0.0)
45	41 (3.3)	1 (1.7)	4 (3.6)	0 (0.0)
51	74 (6.0)	2 (3.4)	**13 (11.6)**	2 (2.7)
52	**295 (23.9)**	**16 (27.6)**	**35 (31.3)**	12 (16.0)*
56	49 (4.0)	2 (3.4)	4 (3.6)	3 (4.0)
58	**194 (15.7)**	**12 (20.7)**	**20 (17.9)**	22 (29.3)**
59	29 (2.4)	2 (3.4)	2 (1.8)	0 (0.0)
66	53 (4.3)	**6 (10.3)**	4 (3.6)	3 (4.0)
68	105 (8.5)	4 (6.9)	8 (7.1)	0 (0.0)**

SIL, squamous intraepithelial lesion; NILM, negative for intraepithelial lesion or malignancy; ASC-US, atypical squamous cells of undetermined significance; LSIL, low-grade squamous intraepithelial lesion; HSIL, high-grade squamous intraepithelial lesion.

***P < 0.05 *vs.* cervicitis/NILM, ASC-US and LSIL groups; **P < 0.05 *vs.* cervicitis/NILM and LSIL groups; *P < 0.05 *vs.* cervicitis/NILM group. Percentages >10 are shown in bold.

### hrHPV prevalences grouped by CIN status

Women with ASC-US (*n* = 58), LSIL (*n* = 112) and HSIL (*n* = 75) underwent colposcopy and biopsy. Ninety-four histological examinations were completed. When determining the prevalences of hrHPVs by CIN status, we grouped women with CIN II, CIN II–III and SCC as CIN II^+^. Fifty-two and 42 women were of CIN I and CIN II^+^ status, respectively (Table 3). As was true for the SIL grouping, the HPV 16 positivity rate was significantly higher among women of CIN II^+^ status (61.9%) than among those with cervicitis (18.0%) and those of CIN I status (21.2%); HPV 68 was not detected in the 42 women of CIN II^+^ status. Although the rates of infection with genotypes 52 and 58 were thus similar to those revealed by SIL grouping, positivity did not differ significantly among the cervicitis/NILM, CIN I and CIN II^+^ groups.

**Table 3.** Prevalences of hrHPVs in patients grouped by CIN status

Genotype	Cervicitis/NILM	CIN I	CIN II^+^
	(*N* = 1234)	(*N* = 52)	(*N* = 42)
16	**222** (**18.0)**	**11** (**21.2)**	**26** (61.9)**
18	**153 (12.4)**	**7 (13.5)**	**2 (4.8)**
31	101 (8.2)	4 (7.7)	3 (7.1)
33	79 (6.4)	**10 (19.2)**	2 (4.8)
35	58 (4.7)	2 (3.8)	0 (0.0)
39	73 (5.9)	2 (3.8)	0 (0.0)
45	41 (3.3)	3 (5.8)	0 (0.0)
51	74 (6.0)	3 (5.8)	1 (2.4)
52	**295 (23.9)**	**16 (30.8)**	**8 (19.0)**
56	49 (4.0)	0 (0.0)	1 (2.4)
58	**194 (15.7)**	**8 (15.4)**	**9 (21.4)**
59	29 (2.4)	1 (1.9)	0 (0.0)
66	53 (4.3)	4 (7.7)	1 (2.4)
68	105 (8.5)	3 (5.8)	0 (0.0)*

HPV, human papillomavirus; CIN, cervical intraepithelial neoplasia; NILM, negative for intraepithelial lesion or malignancy; SCC, squamous-cell carcinoma.

The virus strains with prevalence of more than 10% are marked by bold.

**P < 0.05 *vs.* cervicitis/NILM and CIN I groups.

### Characteristics of co-infecting hrHPVs

In total, 1144, 278 and 60 women were infected by one, two and three or more strains of HPV, respectively (Table 4). The proportions of women with cervicitis/NILM infected with one, two and three or more strains fell gradually from 85.1% to 68.3%; conversely, the proportions increased significantly from 4.7% to 18.3% in those with HSILs. Notably, the proportions of the five most common HPV genotypes (16, 18, 33, 52 and 58) increased significantly from those infected with single strains to those infected with two and three or more strains.

**Table 4.** Characteristics of hrHPV co-infection

	Single strain	Two strains	Three or more strains	*P* for trend
	*N* = 1144	*N* = 278	*N* = 60	
SIL grouping				
Cervicitis/NILM	974 (85.1)	219 (78.8)	41 (68.3)	0.0003
ASC-US	42 (3.7)	14 (5.0)	3 (5.0)	0.5326
LSIL	74 (6.5)	28 (10.1)	5 (8.3)	0.1081
HSIL	54 (4.7)	17 (6.1)	11 (18.3)	<0.0001
Genotype grouping				
16	190 (16.6)	81 (29.1)	30 (50.0)	<0.0001
18	118 (10.3)	47 (16.9)	16 (26.7)	<0.0001
33	58 (5.1)	38 (13.7)	9 (15.0)	<0.0001
52	225 (19.7)	99 (35.6)	31 (51.7)	<0.0001
58	165 (14.4)	57 (20.5)	26 (43.3)	<0.0001

NILM, negative for intraepithelial lesion or malignancy; ASC-US, atypical squamous cells of undetermined significance; LSIL, low-grade squamous intraepithelial lesion; HSIL, high-grade squamous intraepithelial lesion.

## Discussions

We present community-based HPV and Pap co-test baseline data. The overall prevalence of all 14 HPV genotypes was 10.0%, consistent with other data from eastern Asia [3, 4]. We found that the HPV 16 and 58 positivity rates were significantly higher in women with HSILs than in those with cervicitis/NILM and those with LSILs. These distributions were not fully confirmed in the analysis by CIN grade, perhaps due to the small numbers evaluated in terms of CIN status. However, a Chinese study revealed significantly higher rates of HPV 16 and 58 positivity in 170 women of CIN II–III status and 35 women with invasive SCC [17]. A meta-analysis of the worldwide prevalence of HPV 58 in subjects with CIN also showed that the association of HPV 58 with CIN and invasive SCC was 3.7–4.9-fold higher in eastern Asia than elsewhere [18]; these data thus support our conclusion that HPV 16 and 58 are the two predominant hrHPVs in Shanghai.

The HPV 18, 52 and 68 infection rates were significantly lower in women with HSILs than in those with NILM and LSILs. HPV 18 is more strongly linked to adenocarcinoma than is SCC [2, 19]; thus, HPV 18 infection is understandably rare in women with HSILs. HPV 52 has been found to be more prevalent in Chinese outpatients and community-dwelling women [16, 17, 20, 21]. Surprisingly, the HPV 52 and 68 infection rates were significantly lower in women with HSILs; which should be given more attention in future prospective studies. Infection with multiple HPV strains is common, but the potential associations with cervical cancer have not been well studied. We found that the proportion of co-infection was high in women with HSILs, and the proportions of infection with the five most common genotypes (16, 18, 33, 52 and 58) increased from those among women infected with single strains to those infected with three or more strains. Herrero *et al*. [22] showed that infection with multiple HPV strains was associated strongly with increased risks of low-grade lesions and CIN II+ status. Thus, multiple-genotype infection may play a role in the development of cervical cancer.

The clinical sensitivity of hrHPV DNA testing in terms of the detection of CIN II+ status is approximately 95% in screening populations [23, 24], encouraging hrHPV DNA testing by gynaecologists to reduce the proportions of females with equivocal cytology after HPV detection [25]. However, hrHPV distribution patterns vary among countries; definition of the hrHPV pattern in China is thus essential. A total of 1479 HPV-positive women underwent cytological examination, but histological data were available for only 94 of these women. Exfoliative cytological assessments of transformation zones sometimes predict the cancer risk even when histopathological evaluation of colposcopic biopsy material does not confirm the presence of precancerous lesions [26]. Thus, we believe that the distribution characteristics derived by SIL grouping faithfully reflect the relationship between HPV genotype and the extent of cervical lesions.

Most HPV infections are cleared/suppressed by host cell-mediated immune processes within 1–2 years [27]. The most common HPV genotypes should be those capable of persistent infection, as prevalence equals incidence multiplied by duration [2]. Although our study was cross-sectional in nature, and we thus lacked data on persistence, the work suggests that the high prevalence of HPV 16 and 58 reflects persistent infection and hence associated risks of cervical cancer development.

More and more studies confirm that vaccinating adolescents before sexual debut has a substantial impact on the overall incidence of high-grade cervical abnormalities [28–30]. Currently, there are three licensed HPV vaccines, they are bivalent vaccine, which includes HPV genotypes 16 and 18 VLPs; quadrivalent vaccine, which includes HPV genotypes 6, 11, 16 and 18 VLPs; and nine-valent vaccine, which includes HPV genotypes 6, 11, 16, 18, 31, 33, 45, 52 and 58 VLPs [31, 32]. Obviously, nine-valent HPV vaccine is the best choice for local females, because only this vaccine includes both HPV genotypes 16 and 58 VLPs. In addition, in order to promote the efficiency of vaccination, the following issues must be seriously explored and/or monitored in China [30]: HPV epidemic spectrum before and after vaccination; population coverage of HPV vaccination; optimal target age range for vaccination; immunisation of males and herd effects; and incidence of cervical cancer.

In conclusion, HPV 16 and 58, but not 16 and 18, are associated with local women with HSIL; the HPV 18, 52 and 68 infection rates were significantly lower in local women with HSIL; and the proportion of women infected by multiple HPV strains was higher in local women with HSIL.
